# Personal

**Published:** 1900-07

**Authors:** 


### PERSONAL.

---

MAJOR A. H. BRIGGS, M.D., of Buffalo, Surgeon 65th Regt., N.Y. S.N.G., attended the recent meeting of the Association of Military Surgeons of the United States at New York City. Dr. Briggs is an active member of the Association, was one of the founders, and enjoys the confidence and esteem of the association. He delivered the address of welcome at the meeting, and took a prominent part in its proceedings.

---

DR. ABRAM TUCKER KERR, of Buffalo, a graduate of Cornell in the class of 1895, and of the Medical Department of the University of Buffalo in 1897, has been appointed assistant professor of anatomy, to fill the recently created chair at Cornell University. This will be a distinct loss to Buffalo, and as distinct a gain to Cornell.

---

DR. MARCEL HARTWIG was elected president of the Buffalo Academy of Medicine at the last annual meeting. Dr. Hartwig signaled his election by offering a prize of $50, cash or value, for the best essay on a scientific medical subject by a member of the Academy, to be presented during the academic year.

---

DR. SAMUEL G. GANT, of Kansas City, Mo., recently elected professor of rectal and anal surgery in the New York Post-Graduate Medical School and Hospital, has removed to No. 58 West 56th Street, New York City.

Dr. F. W. McGuire, of Buffalo, was elected secretary of the section on pathology of the Academy of Medicine, at the recent annual meeting of the section.

---

Dr. Abraham Jacobi, of New York, president of the Medical Board of Mount Sinai Hospital, lately received a silver tankard as a gift from the Medical Board and the 4,200 members of the hospital on the occasion of the seventieth anniversary of his birth. The presentation was made by President Wallach of the hospital, who spoke of the service Dr. Jacobi had rendered the hospital for forty years. Dr. Jacobi made an appropriate response. Dr. Jacobi, who is professor of the diseases of children in the College of Physicians and Surgeons, also received the degree of LL.D., at the recent annua commencement of Columbia University.

---

Dr. Eugene A. Smith and Dr. Nelson G. Russell, of Buffalo, have been appointed on the staff of visiting physicians at the Fresh Air Mission Hospital at Athol Springs for this season.

---

Dr. S. Goldberg, of Buffalo, will sail on the "St. Louis," July 4th, for several months recreation and study in Europe.

---

Dr. Eugene A. Smith,, of Buffalo, was elected chairman of the section on pathology, Buffalo Academy of Medicine, at the meeting held June 19, 1900.

---

Dr. Ernest Wende, of Buffalo, was elected chairman of the section on State Medicine at the recent meeting of the American Medical Association at Atlantic City. On his return from this meeting Dr. Wende fell ill with appendicitis, but happily has made a prompt recovery, and is again attending to his professional and official duties.

